# Modeling Temperature Requirements for Growth and Toxin Production of *Alternaria* spp. Associated with Tomato

**DOI:** 10.3390/toxins17080361

**Published:** 2025-07-23

**Authors:** Irene Salotti, Paola Giorni, Chiara Dall’Asta, Paola Battilani

**Affiliations:** 1 Department of Sustainable Crop Production (DI.PRO.VE.S.), Università Cattolica del Sacro Cuore, 29122 Piacenza, Italy; irene.salotti1@unicatt.it (I.S.); paola.giorni@unicatt.it (P.G.); 2 Department of Food and Drug, University of Parma, Parco Area delle Scienze 17/A, 43124 Parma, Italy; chiara.dallasta@unipr.it

**Keywords:** *Solanum lycopersicum*, mycotoxin, alternariol, alternariol monomethyl ether, tenuazonic acid

## Abstract

Concerns about mycotoxin contamination by *Alternaria* spp. in tomato-based products emphasize the need for understanding the effect of the environment on their production. In the current study, we focused on three species frequently associated with tomato (*A. alternata*, *A. solani*, and *A. tenuissima*) by evaluating the effects of different temperatures (5 to 40 °C) and substrata (PDA and V8) on mycelial growth and the production of mycotoxins (alternariol, alternariol monomethyl ether, and tenuazonic acid). Both biological processes were supported between 5 and 35 °C, with optimal temperatures between 20 and 30 °C, depending on the species. Temperature and its interaction with species significantly (*p* < 0.05) affected both processes. However, the species factor alone was not significant (*p* > 0.05), indicating that environmental conditions affect *Alternaria* spp. growth and mycotoxin production more than the species itself does. Mathematical equations were developed to describe the effect of temperature on mycelial growth, as well as on the production of AOH, AME, and TeA, for each *Alternaria* species. High concordance (CCC ≥ 0.807) between observed and predicted data and low levels of residual error (RMSE ≤ 0.147) indicated the high goodness of fit of the developed equations, which may be used for the development of models to predict *Alternaria* contamination both in field and during post-harvest storage.

## 1. Introduction

Several species within the genus *Alternaria* have been frequently reported as tomato (*Solanum lycopersicum* L.) pathogens able to cause early blight epidemics and fruit rot in field, as well as post-harvest decay [1,2,3]. Furthermore, *Alternaria* species are well known as mycotoxigenic fungi that can contaminate food products and pose health risks to humans [4,5].

Although the taxonomic revision of *Alternaria* spp. has frequently occurred and is still an ongoing process that can hamper or complicate species identification, more than 790 species epithets and approximately 368 species grouped into 29 sections are currently recognized based on their morphology and molecular phylogeny [6]. Both small-spored species of *Alternaria* section, such as *A. alternata* and *A. tenuissima*, and large-spored species of *Porri* section, such as *A. solani*, have been commonly associated with tomato and have been isolated from diseased leaves and fruit, as well as from contaminated tomato-based products. For example, a study conducted in northern Italy between 2017 and 2019 revealed that *A. alternata* was present in 75% of symptomatic tomato samples, while *A. tenuissima* was found in the remaining samples [7]. In another study carried out in southern Italy, *A. alternata* and *A. tenuissima* were isolated from 60% and 20% of samples, respectively, [8]. Additionally, *A. solani* has been historically associated with early blight, and large-spored *Alternaria* species found on Solanaceous plants have often been determined as *A. solani* [9].

*Alternaria* species can directly infect tomato plants and produce a wide range of enzymes and toxins (both host-specific and nonspecific) that play a key role in plant pathogenesis and facilitate inner tissue colonization and the extraction of nutrients. The most common toxins belong to the following structural groups: dibenzopyrone derivatives such as alternariol (AOH) and alternariol monomethyl ether (AME); perylene derivates such as altertoxins (ATX-I, ATX-II, and ATX III); and tetramic acid derivatives such as tenuazonic acid (TeA) [10,11]. AOH and AME are virulence factors that cause necrotic lesions via a largely unknown mode of action; the former probably induces mitochondrial apoptosis, while the latter inhibits photosynthetic activity [12]. On the other hand, TeA is known for its ability to inhibit photosystem II in plants [13].

Because AOH, AME, and TeA are produced from the early stages of the *Alternaria* spp. infection cycle, they accumulate within plant tissues during epidemic development and can then contaminate tomatoes and tomato-based products along the food chain [4], posing a threat to human and animal health. In fact, AOH, AME, and TeA have been reported to cause mutagenic, estrogenic, and clastogenic effects in microbial and mammalian cell systems [14,15]. TeA and AOH are the most common mycotoxins found in retail tomato products, as demonstrated by several surveys carried out in Europe and South America [7,8,16,17,18,19,20,21]. For instance, TeA was the main contaminant observed in Belgian, Swiss, Brazilian, and Argentinian tomato products, with an incidence ranging from 18% to 100% (with 18 to 85 samples in the surveys) and concentrations from a few ng/kg to up to more than 4000 μg/kg. At lower frequencies or concentrations, AME and AOH were also found in these samples [7,8,17,18,20,21]. However, AOH was observed in over 93% of samples of German tomato products, with a detection limit of 1–13 μg/kg [16]. Conjugated mycotoxins such as AOH-3-sulfate and AME-3-sulfate as well as native forms were also observed in several studies [19,20]. Legal limits on the maximum concentration of *Alternaria* toxins in tomato-derived products, however, are not currently available. Nonetheless, the European Food Safety Authority (EFSA) performed a risk assessment in 2016 and established a toxicological concern threshold for AOH and AME of 2.5 ng/kg bw per day and for TeA of 1500 ng/kg bw per day [15]. Then, in 2022, the European Union published the Commission Recommendation (EU) 2022/553 of 5 April 2022 on Monitoring the Presence of *Alternaria* Toxins in Food [22], which included indicative limits for *Alternaria* toxins in processed tomato products, i.e., 5, 10, and 500 μg/kg for AOH, AME, and TeA, respectively.

Despite the importance of *Alternaria* mycotoxins in tomato, the lack of knowledge on the environmental conditions influencing their production was reported in a recent review [23]. Only Pose et al. [24] and Vaquera et al. [25] studied the effect of temperature and water activity on *Alternaria* species associated with tomato. The former focused on *A. alternata*, testing only four temperatures (6, 15, 21, and 35 °C) and water activity (aw) values (0.904, 0.922, 0.954, and 0.982), while the latter studied *A. arborescens*, which is reported as only associated with tomato stem canker.

To address the limited information available in the literature, in the current study, we aim to investigate temperature requirements (in the broad range of 5 to 40 °C) for mycelial growth and the production of mycotoxins (AOH, AME, and TeA) in three species of *Alternaria* well known to affect tomato leaves and fruits, i.e., *A. alternata*, *A. solani*, and *A. tenuissima*.

## 2. Results

### 2.1. Mycelial Growth

According to analysis of variance (ANOVA) results, significant (*p* < 0.01) factors affecting mycelial growth were temperature, days post-inoculation, and their interaction, which contributed to 54%, 35%, and 6% of the variance, respectively. Growth media did not have a significant impact; therefore, mean growth data were considered for data analysis.

**Figure 1:**
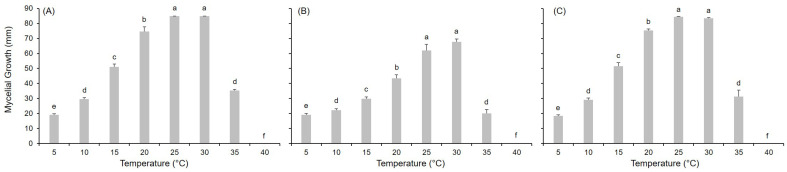
Fungal colony diameter (mm) reached after 14 days of incubation at the reported temperature for (**A**) *Alternaria alternata*, (**B**) *A. solani*, and (**C**) *A. tenuissima*. Bars represent the average growth on PDA and V8 in two experiments; whiskers represent the standard error. Different letters define significant difference according to Fisher’s Least Significant Difference (LSD) test at α = 0.05.

Growth was observed for all the *Alternaria* species between 5 °C and 35 °C but not at 40 °C (Figure 1). At the optimum temperature of 25–30 °C, the fungal colonies reached the maximum diameter (i.e., 86 mm) within 12 to 14 days. A temperature of 20 °C was recorded as sub-optimal for mycelial growth in all species. Intermediate levels of growth were guaranteed at 15 °C, followed by those at 10 °C and 35 °C. Mycelial growth was poorly supported at 5 °C, even after 14 days (<24% of maximum recorded growth).

**Figure 2:**
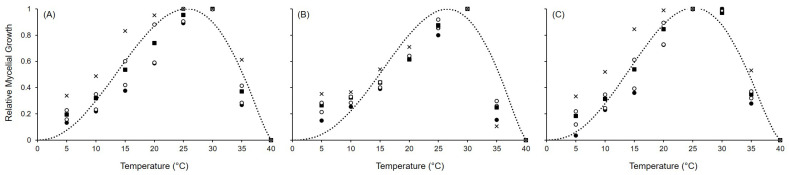
Mycelial growth for (**A**) *Alternaria alternata*, (**B**) *A. solani*, and (**C**) *A. tenuissima*. Symbols show the average growth on PDA and V8 in two experiments after 5 (•), 7 (∘), 12 (■), 14 (□), and 21 (x) days at the reported temperature. The dotted lines show the fit of data using Equation (1); equation parameters for each species are summarized in Table 1.

Equation (1) provided a good fit for the mycelial growth data when 0 °C and 40 °C were used as the minimum and maximum temperatures, respectively, with the coefficient of determination adjusted for a degree of freedom (R^2^) ≥ 0.807 and concordance correlation coefficient (CCC) values ranging between 0.898 and 0.948 (Figure 2; Table 1). A small average distance between the real data and the fitted line was observed, with a root mean square error (RMSE) ≤ 0.147. The coefficient of residual mass (CRM) from 0.021 to 0.053 indicated a slight tendency toward underestimation. Estimates for the model parameters were between 5.975 and 6.655 for *a*, 1.703 and 1.969 for *b*, and 1.297 and 1.485 for *c*, showing similar responses to temperature in all the three species, with an estimated optimal temperature (Topt) of 25.6 °C, 26.5 °C, and 25.2 °C for *A. alternata*, *A. solani*, and *A. tenuissima*, respectively.

### 2.2. Mycotoxin Production

According to the ANOVA results, temperature and its interaction with species were significant (*p* < 0.05) factors affecting the production of mycotoxins, while the type of mycotoxin produced (AOH, AME, or TeA) was poorly significant (*p* = 0.0526).

Nevertheless, further analyses of variance were performed separately for each mycotoxin to better investigate the effect of temperature and species on their production.

Temperature and its interaction with species were the only significant (*p* < 0.05) factors affecting AOH, AME, or TeA, when singularly considered in ANOVA. Temperature exerted a major effect, explaining 79%, 85%, and 87% of the total variance for AOH, AME, and TeA production, respectively. Meanwhile, the interaction between temperature and species explained only 7%, 8%, and 12% of the total variance for AOH, AME, and TeA production, respectively, and the species factor alone did not significantly affect the production of AOH (*p* = 0.13), AME (*p* = 0.17), or TeA (*p* = 0.40).

Overall, optimal temperatures ranged between 20 °C and 30 °C, depending on the species. Mycotoxin production decreased at 15 °C and 35 °C, followed by 10 °C. No or negligible (≤0.3% of maximum concentration on average) production was observed at 5 °C (Table 2). The highest production of AOH (1251 ± 74.8 μg/kg) was observed at 25 °C in *A. solani*, for which the range of optimum temperature was between 20 °C and 30 °C. Instead, narrower ranges of optimum temperature were observed for *A. alternata* and *A. tenuissima*, with a shift toward higher (25–35 °C) and lower (15–25 °C) temperatures, respectively. Concerning AME, high production (≥530 μg/kg) was detected in *A. solani* at 25 °C and 30 °C and in *A. alternata* between 25 °C and 35 °C. Meanwhile, *A. tenuissima* achieved maximum production at 20 °C, but this was lower (412.0 ± 25.9 μg/kg) compared to that reached by other species. On the contrary, *A. tenuissima* achieved the highest TeA production at temperatures between 10 °C and 25 °C compared to the other species, with maximum production (721.2 ± 71.6 μg/kg) at 25 °C. Only at 30 °C and 35 °C was TeA production higher in *A. alternata* and *A. solani*.

**Figure 3:**
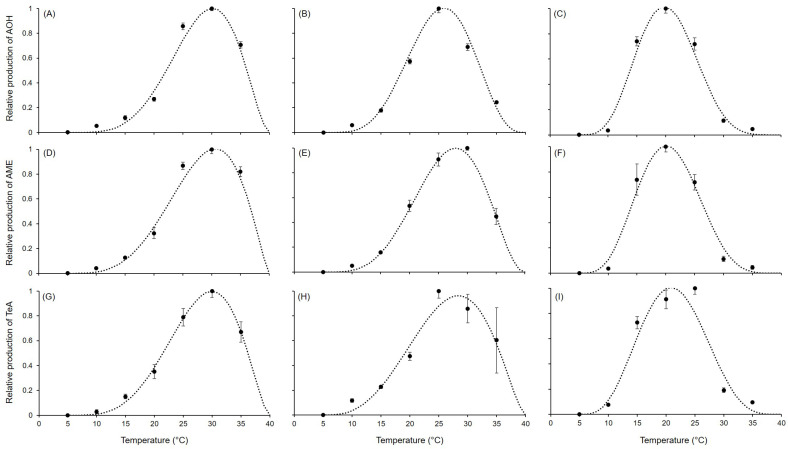
Production of different mycotoxins in (**A**,**D**,**G**) *Alternaria alternata*, (**B**,**E**,**H**) *A. solani*, and (**C**,**F**,**I**) *A. tenuissima*. Symbols show the average production on PDA and V8 in two experiments after 21 days at the reported temperatures; whiskers represent the standard error. The dotted lines show the fit of data using Equation (1); equation parameters for each species are summarized in Table 3.

Equation (1) provided a good fit for the AOH, AME, and TeA data for all *Alternaria* species (Figure 3; Table 3), with R^2^ ≥ 0.922 and CCC ranging from 0.966 to 0.999. Low RMSE values (from 0.02 to 0.102) were obtained. A slight tendency toward underestimation (CRM from 0.011 to 0.024) was observed, except for in the fit of AOH and TeA in *A. tenuissima*, which showed slight overestimation (CRM of −0.01 and −0.002, respectively). Estimates for the equation parameters ranged from 3.252 to 8.833 for *a*, from 0.755 to 2.748 for *b*, and from 1.386 to 5.832 for *c*. A similar pattern in the production of AOH, AME, and TeA was observed within the same species, as reflected by the closeness of estimated values of *a*, *b*, and *c* among the mycotoxins and the species (Figure 2). In fact, *A. alternata* had higher values in *a* and *b* and lower values in *c* compared to the other species, which resulted in wider, left-skewed temperature–response curves, indicating that all the mycotoxins were abundantly produced at higher temperatures. In fact, the calculated optimum temperatures (Topt in Table 3) were 30.1 °C, 30.7 °C, and 30.0 °C for AOH, AME, and TeA, respectively. On the contrary, lower values in *a* and *b* and higher *c* values were obtained for *A. tenuissima*, which presented symmetrical temperature-dependent curves with peaks (corresponding to Topt) at 19.8 °C, 20.1 °C, and 20.9 °C for AOH, AME, and TeA, respectively. For *A. solani*, the parameters assumed estimated values in between those of *A. alternata* and *A. solani*, resulting in temperature-dependent curves showing slightly asymmetry for AOH (with Topt = 25.9 °C) and left-skewed distributions for AME (Topt = 28.0 °C) and TeA (Topt = 28.4 °C).

## 3. Discussion

Many species of *Alternaria* can produce *Alternaria* toxins, even if to a different extent and under environmental conditions that can be greatly influential. Climate may play an important role in these species’ production of mycotoxins, in particular water activity (aw) and temperature [26].

Although different species of *Alternaria* can be found in tomato fields, only a few studies on ecological requirements for the production of toxins have been conducted and many of them have taken only a limited number of *Alternaria* species into account. In particular, only *A. alternata* and *A. arborescens* were previously used for in vitro studies to define the production of *Alternaria* toxins under different patterns of temperature and aw levels [25,27], while in other studies considering *A. solani* and *A. tenuissima*, only restricted ecological conditions were taken into account [28,29].

In this study, temperature was considered as the predominant environmental factor affecting mycelial growth and mycotoxin production in tomato crops. The effect of water activity was not investigated because it does not represent a limiting factor in tomato, whose tissues fulfills water requirements for fungal development. Research gaps identified by Salotti et al. [23] were addressed, especially regarding *A. tenuissima*. Available knowledge on three *Alternaria* species frequently associated with leaf and fruit diseases in tomato, i.e., *A. alternata*, *A. solani*, and *A. tenuissima*, was enlarged by broadening the temperatures tested for evaluating mycelial growth and mycotoxin production on two substrates, a standard medium (PDA) and one simulating tomato berries (V8); no significant impact on fungal behavior was shown under these study conditions.

In this study, *A. alternata* was able to produce the highest amounts of all mycotoxins tested (AOH, AME, and TeA) at 25–35 °C. This is in agreement with previous reports, in which *A. alternata*’s maximum capacity to produce different toxins was established at 25 °C and a high level of aw (>0.98 aw) [30,31].

The ecological needs for the production of toxins by *A. solani* seem to be similar to those of *A. alternata* with only a slight tendency to require a lower temperature range (20–25 °C) instead of 25–30 °C. In a previous paper, it was indicated that the same strain of *A. solani* did not produce TeA [28], while in this study, the production of this toxin was not negligible. This was probably due to the different experimental conditions, the age of the colonies used for mycotoxin assessment (which were 7 days older in the current study), or the mycotoxin extraction method.

Interesting results were obtained for *A. tenuissima*, which showed similar trends to those for *A. solani* and *A. alternata* regarding AME and TeA, but the highest production of AOH was found in a lower temperature range (15–20 °C). This could indicate that this species possibly plays a main role in tomato contamination in the case of adverse climatic conditions during field cultivation.

Similarly to those for mycotoxin production, the mycelial growth results confirmed most previously reported findings (e.g., [32,33,34,35,36,37]), which concerned one or more isolates and reported optimal temperatures between 25 and 30 °C and the ability to grow at temperatures as low as 5 °C. However, no mycelial growth in A. *alternata* and *A. solani* was detected at 40 °C, whereas this was reported by other authors [33]. A maximum temperature of 40 °C was also found for *A. tenuissima*, which had not previously been tested [32].

Given similarities in temperature requirements and lack of significant difference among the three species in mycelial growth and mycotoxin production, the current work suggests that environmental conditions affect both biological processes more than *Alternaria* species. Although more studies are necessary to confirm this result with a greater number of species and isolates, these findings provide insight into the management of *Alternaria* diseases on leaves and fruits, as well as future scenarios involving their occurrence in relation to climate change.

It is worth noting that all three species can grow and produce mycotoxins over a wide temperature range; however, the best conditions are associated with warm temperatures, i.e., those above 25 °C, with the only exception of *A. tenuissima*, which achieved maximum mycotoxin production at 20 °C. Recently, Delgado-Baquerizo et al. [38] reported *Alternaria* as the most common pathogenic fungal genus found in a global soil sampling survey and tested the effect of global warning (∼2 °C) on its abundance. Under these climate change projections, warming doubled the abundance of *Alternaria* in most regions of the world, regardless of the climate and land use scenarios considered. Another simulation study by Van de Perre et al. [39] specifically focused on the effect of climate change on *A. arborescens* growth and mycotoxin production in tomatoes. The predicted higher temperature in the near (2031–2050) and far future (2081–2100), which will become closer to the optimal temperature for the growth of *Alternaria* spp., will lead to more severe damage and mycotoxin contamination in tomato, including in new areas. For instance, their simulation highlighted the spread of these problems in Poland in the far future due to warming compared with the colder temperatures in the present. The knowledge acquired in the current study may help to support similar climate change simulation studies, specifically those focusing on *A. alterntata*, *A. solani*, or *A. tenuissima*, as well as climate matching analysis regarding the spread of these pathogens in new areas.

Information on the temperature requirements for the development of *Alternaria* species is needed to support disease management and ensure food safety. Several contemporary plant disease prediction systems are developed using a mechanistic approach, which depicts the relationships between the pathogen, host, and environment and quantifies the effects of weather factors on the development of epidemics and/or product contamination [40,41]. The prediction of an event, days or weeks before its occurrence, allows growers to respond in a timely and efficient manner by adjusting crop management practices. Moreover, the prediction of a low risk of contamination may result in the reduced application of plant protection products, improving farm sustainability [42,43].

Although *Alternaria* diseases have historically been described as singleton species that cause early blight or brown spots, recent insights revealed the concurrent presence of several species in tomato plants, even with different genotypes on the same lesion. Consequently, the hypothesis emerged that several species are involved in a disease complex [44]. The equations developed in this study help to identify similarities and differences in the effects of temperature on *Alternaria* species comprised in this complex. The high concordance (CCC ≥ 0.807) between the observed and predicted data and the low levels of residual error (RMSE ≤ 0.147) indicate that the developed equations reliably represent the effect of temperature on both mycelial growth and the production of three different mycotoxins, i.e., AOH, AME, and TeA. The developed equations may also be of value in predicting contamination by *Alternaria* both in field epidemics and during post-harvest storage. Mycotoxin contamination has often been predicted in terms of contamination risk in several modeling approaches and pathosystems. The equations proposed in the current work particularly fit this aim because they describe how mycotoxigenic fungi respond to temperature, a key driver of the mycotoxin production process. Rather than absolute quantities (which may depend on several factors beyond those investigated in the current study), the equations provide values from 0 to 1, showing the scattering of the response to temperature on a rational scale. This provides an initial overview of favorable conditions for mycotoxin contamination, supporting decisions to prevent or manage possible mycotoxin contamination under ongoing conditions. Furthermore, the temperature-dependent response alone, or that accumulated over a period (e.g., days of the epidemic), can be used in comparison with real observation datasets to draw a probabilistic relationship between real mycotoxin contamination and define a contamination risk level related to overcoming legal or recommended limits, as in the case of *Alternaria*. Thus, it can be included in plant disease models and warning systems to support tactical decisions for sustainable mycotoxin management.

## 4. Materials and Methods

### 4.1. Fungal Isolates and Culture Conditions

Three single-spore strains of *Alternaria alternata*, *A. solani*, and *A. tenuissima* were obtained from the culture collections of Westerdijk Fungal Biodiversity Institute, the Netherlands (Table 4). The isolates had different geographic origins and were obtained from symptomatic *Solanum* plants and/or have been reported as pathogenic on *S. lycopersicum* [45]. The strains were grown on potato dextrose agar (PDA) at 42 g L^−1^ (HiMedia Laboratories, Mumbai, India) at 25 °C, with a 12 h photoperiod, for two weeks before use. The three isolates were used in the mycelial growth and mycotoxin production experiments.

### 4.2. Mycelial Growth

Mycelial plugs (4 mm in diameter) were obtained using a cork borer from 2 to week-old colonies and placed individually in the center of new Petri dishes (8.6 cm in diameter) containing PDA or V8 (HiMedia Laboratories, Mumbai, India; 44.3 g L^−1^). The inoculated Petri dishes were incubated at 5, 10, 15, 20, 25, 30, 35, or 40 °C in darkness. Three replicate plates for each combination of strain and temperature were assessed after 5, 7, 12, 14, and 21 days. Two perpendicular diameters of each colony were measured and used to calculate their average diameter. The relative mycelial growth was then calculated by dividing the average colony diameter by the diameter of the Petri dish. The experiment was performed twice.

### 4.3. Mycotoxin Analysis

After 21 days of growth, the cultures were frozen and stored at −18 °C until mycotoxin analysis.

#### 4.3.1. Reagents and Chemicals

LC/MS-grade methanol (MeOH), acetonitrile (MeCN), ultrapure water, formic acid (FA), and acetic acid were obtained from VWR Chemicals (VWR International, Milan, Italy). Commercially available analytical standards of alternariol (AOH), alternariol monomethyl ether (AME), and tenuazonic acid (TeA) were purchased from Romer Labs (Tulln, Austria). Working solutions of 1 μg L^−1^ were prepared in acetonitrile and further diluted to obtain matrix-matched calibration curves for quantification. The working solutions were stored at −20 °C and prepared freshly every month.

#### 4.3.2. Mycotoxin Extraction

After lyophilization and grinding, a 0.5 g sample was weighted into a 15 mL tube and extracted with 2 mL of acetonitrile/water (80/20, *v*/*v*) mixture acidified with 0.2% of formic acid. The sample was stirred for 90 min at 200 strokes/min using a shaker and then centrifuged for 10 min at 14,000 rpm. The supernatant (1 mL) was evaporated to dryness and redissolved in the mobile phase (5% eluent B, under the initial conditions). After filtration using 0.2 μm filters, 1 μL of the supernatant was injected into LC–MS.

#### 4.3.3. UHPLC-TWIMS-QTOF Screening of Mycotoxins

The analysis was performed as reported by Rabaaoui et al. [46]. The quantification of the target analytes was performed using calibration standards in the range 1–200 μg kg^−1^, setting the LOQ at 1 μg kg^−1^ for the 3 analytes. Samples exceeding the calibration range were properly diluted.

### 4.4. Statistical Analysis

All the analyses were performed using R software v. 4.3.2 (R Core Team. R: A Language and Environment for Statistical Computing 2023; available at https://www.r-project.org/, accessed on 31 October 2023).

Statistical data analysis was carried out by applying analysis of variance (ANOVA) and Fisher’s Least Significant Difference (LSD) post hoc test (α = 0.05) to investigate the influence of *Alternaria* species, temperature, and substrata on the growth of mycelium and the production of mycotoxins, i.e., AOH, AME, and TeA. The time factor (expressed as the number of days after the inoculation of the substrata) was also included in the ANOVA for the evaluation of mycelial growth. Mycelial growth and the mycotoxin production were transformed by the natural logarithm (y = ln(x + 1)) to homogenize the variance, before applying the ANOVA test. Variance homogeneity was then confirmed by applying the Bartlett test (*p* = 0.05) after data transformation, using the function bartlett.test in the ’stats’ package. The ANOVA was performed using the function aov in the ‘stats’ package, while the LSD test was performed using the function LSD in the ‘agricolae’ package.

Temperature-dependent equations for the growth of mycelium and the production of each mycotoxin were developed. Data were first rescaled to the highest number found in the experiment (at the optimal temperature for growth or mycotoxin production) and then regressed against temperature using different non-linear regression equations. The best equation was selected based on Akaike’s information criterion (AIC). The Analytis Equation [47] provided the smallest AIC values and was therefore considered the most likely to be correct [48].

The Analytis [47] equation was used in the following form:

(1) $$Y={\left[a\times Teq^{b}\times \left(1-Teq\right)\right]}^{c}$$

where *Y* is the rescaled value of mycelial growth or mycotoxin production (on a 0–1 scale). *Teq* is the temperature equivalent, defined as follows:

(2) Teq=(T−Tmin)/(Tmax−Tmin)

where *T* is the temperature regime (in °C) and *Tmin* and *Tmax* are the minimal and maximal temperatures that support the process (in °C); *a*, *b*, and *c* are the equation parameters, defining the top, symmetry, and size of the bell-shaped curve, respectively.

The optimal temperature (*Topt* in °C) for mycelial growth and the production of each mycotoxin was then calculated as follows:

(3) Topt=[(b×c)/(b×c+c)]×(Tmax−Tmin)+Tmin

The parameters for Equation (1) were estimated using the function nls in the ‘stats’ package. The goodness of fit was estimated based on the adjusted R^2^ value, the root mean square error (RMSE), the coefficient of residual mass (CRM), and the concordance correlation coefficient (CCC). In brief, the RMSE is the average distance of real data from the fitted line and was obtained using the rmse function in the R ‘modelr’ package [49]. CRM is a measure of the tendency of an equation to overestimate or underestimate the observed values (a negative CRM indicates the tendency of the model towards overestimation; [50]). CCC is the product of two terms: it is the Pearson product–moment correlation coefficient between observed and predicted values and the coefficient Cb, which indicates the difference between the best-fitting line and the perfect agreement line (if CCC = 1, the agreement is perfect; [51]). The CCC was obtained using the CCC function in the ‘DescTools’ package [52].

## Figures and Tables

**Table 1:** Cardinal temperatures (in °C; used in Equation (2)), calculated optimum temperature (in °C; Equation (3)), estimated parameters (*a*, *b*, and *c*) with their standard errors (in parenthesis), and indexes of the goodness of fit from non-linear regression analysis (Equation (1)) developed to calculate the effect of temperature on the mycelial growth of *Alternaria alternata*, *A. solani*, and *A. tenuissima*.

Species	Tmin	Tmax	Topt	*a*	*b*	*c*	R^2^	CCC	RMSE	CRM
*A. alternata*	0	40	25.6	6.131 (0.342)	1.771 (0.108)	1.297 (0.183)	0.857	0.927	0.132	0.023
*A. solani*	0	40	26.5	6.655 (0.353)	1.969 (0.115)	1.342 (0.262)	0.807	0.898	0.147	0.053
*A. tenuissima*	0	40	25.2	5.975 (0.270)	1.703 (0.082)	1.485 (0.167)	0.897	0.948	0.117	0.021

**Table 2:** Mycotoxin production under different temperature regimes after 21 days of incubation on potato dextrose agar (PDA) and V8. Data are reported as mean and standard error of toxin concentration (μg/kg; three replicates for each substrate and temperature). Different letters define significant difference according to Fisher’s Least Significant Difference (LSD) test at α = 0.05; n.d. stands for not detected (i.e., concentration was lower than limit of detection LOQ = 1 μg/kg for three analytes).

Temperature	*A. alternata*	*A. solani*	*A. tenuissima*
Alternariol (AOH)			
5 °C	2.4 ± 1.1 e	n.d.	1.3 ± 0.7 e
10 °C	56.8 ± 4.5 d	74.0 ± 5.9 cd	26.0 ± 3.6 d
15 °C	124.7 ± 13.8 bcd	221.7 ± 18.2 abcd	528.0 ± 34.2 abc
20 °C	289.3 ± 28.1 abcd	719.5 ± 47.8 ab	712.5 ± 36.1 ab
25 °C	914.8 ± 44.0 a	1251.3 ± 74.8 a	510.3 ± 35.9 abc
30 °C	1067.7 ± 44.1 a	857.5 ± 34.2 ab	79.0 ± 8.2 cd
35 °C	755.2 ± 41.3 ab	303.5 ± 16.5 abcd	32.8 ± 5.9 d
Alternariol monomethyl ether (AME)			
5 °C	2.7 ± 1.5 g	n.d.	1.3 ± 0.9 g
10 °C	27.3 ± 2.9 ef	36.0 ± 4.8 def	14.8 ± 1.8 f
15 °C	81.8 ± 4.9 bcdef	111.0 ± 8.5 abcde	301.0 ± 58.9 abcd
20 °C	215.3 ± 38.9 abcd	376.2 ± 33.1 ab	412.0 ± 25.9 ab
25 °C	562.5 ± 25.9 a	639.3 ± 38.0 a	317.7 ± 16.3 abc
30 °C	646.8 ± 24.4 a	704.5 ± 13.9 a	63.0 ± 9.2 cdef
35 °C	530.0 ± 26.1 a	316.0 ± 49.0 abcd	49.5 ± 7.1 def
Tenuazonic acid (TeA)			
5 °C	n.d.	n.d.	n.d.
10 °C	14.8 ± 9.5 f	45.2 ± 3.6 ef	54.2 ± 6.5 ef
15 °C	80.8 ± 6.5 cdef	91.8 ± 8.0 bcdef	510.0 ± 24.7 abc
20 °C	184.7 ± 20.2 abcdef	192.3 ± 18.6 abcdef	636.7 ± 38.7 ab
25 °C	435.7 ± 31.9 abcd	402.2 ± 29.9 abcd	721.2 ± 71.6 a
30 °C	562.5 ± 52.5 abc	366.5 ± 90.4 abcdef	137.0 ± 18.2 abcdef
35 °C	403.0 ± 87.7 abcde	268.5 ± 169.5 abcdef	69.2 ± 10.4 def

**Table 3:** Cardinal temperatures (in °C; used in Equation (2)), calculated optimum temperature (in °C; Equation (3)), estimated parameters (*a*, *b*, and *c*) with their standard errors (in parenthesis), and indexes of the goodness of fit from non-linear regression analysis (Equation (1)) developed to calculate the effect of temperature on the mycotoxin production of *Alternaria alternata*, *A. solani*, and *A. tenuissima*.

Species	Tmin	Tmax	Topt	*a*	*b*	*c*	R^2^	CCC	RMSE	CRM
Alternariol (AOH)										
*A. alternata*	5	40	30.1	8.237 (0.501)	2.544 (0.155)	1.803 (0.312)	0.979	0.991	0.052	0.019
*A. solani*	5	40	25.9	5.339 (0.187)	1.482 (0.062)	3.231 (0.408)	0.985	0.993	0.041	0.024
*A. tenuissima*	5	40	19.8	3.252 (0.091)	0.728 (0.030)	5.832 (0.811)	0.982	0.992	0.049	−0.010
Alternariol monomethyl ether (AME)										
*A. alternata*	5	40	30.7	8.833 (0.592)	2.748 (0.183)	1.386 (0.246)	0.984	0.993	0.046	0.008
*A. solani*	5	40	28.0	6.522 (0.131)	1.917 (0.041)	2.180 (0.126)	0.997	0.999	0.020	0.011
*A. tenuissima*	5	40	20.1	3.323 (0.131)	0.755 (0.043)	5.288 (0.972)	0.966	0.985	0.066	0.013
Tenuazonic acid (TeA)										
*A. alternata*	5	40	30.0	8.075 (0.228)	2.490 (0.074)	1.640 (0.133)	0.996	0.998	0.023	0.013
*A. solani*	5	40	28.4	6.626 (0.603)	2.016 (0.194)	1.526 (0.373)	0.951	0.979	0.072	0.019
*A. tenuissima*	5	40	20.9	3.529 (0.233)	0.829 (0.077)	4.322 (1.196)	0.922	0.966	0.102	−0.002

**Table 4:** Isolates of *Alternaria* species included in this study, with their country of origin and the substrate from which they were obtained. The isolates were obtained from the culture collections of Westerdijk Fungal Biodiversity Institute, the Netherlands.

Code	Species	Country of Origin	Isolation Substrate
CBS 118814	*A. alternata*	United States of America, Florida, Quincy	*Solanum lycopersicum*
CBS 109157	*A. solani*	United States of America, Washington, Douglas	*Solanum tuberosum*
CBS 117.44	*A. tenuissima*	Denmark, Sjaelland, Clausdal	*Clarkia* spp.

## Data Availability

The original contributions presented in this study are included in the article. Further inquiries can be directed to the corresponding author.
